# A long-term ecological research dataset from the marine genetic monitoring programme ARMS-MBON 2020-2021

**DOI:** 10.3897/BDJ.13.e148981

**Published:** 2025-11-21

**Authors:** Justine Pagnier, Louise Allcock, Ibon Cancio, Eva Chatzinikolaou, Giorgos Chatzigeorgiou, Nathan Alexis Mitchell Chrismas, Federica Costantini, Thanos Dailianis, Klaas Deneudt, Oihane Díaz de Cerio, Markos Digenis, Katrina Exter, Vasilis Gerovasileiou, Jose González Fernández, Laura Kauppi, Kleoniki Keklikoglou, Jon Bent Kristoffersen, Rune Lagaisse, Borut Mavrič, Jonas Mortelmans, Estefania Paredes, Christina Pavloudi, Alessandro Piazza, Anne Marie Power, Andreja Ramšak, Ioulia Santi, Jostein Solbakken, Melanthia Stavroulaki, Peter Anton Upadhyay Stæhr, Javier Tajadura, Jesus Souza Troncoso, Katerina Vasileiadou, Emmanouela Vernadou, Matthias Obst

**Affiliations:** 1 Gothenburg Global Biodiversity Centre, Gothenburg, Sweden Gothenburg Global Biodiversity Centre Gothenburg Sweden; 2 University of Gothenburg, SciLifeLab, Gothenburg, Sweden University of Gothenburg, SciLifeLab Gothenburg Sweden; 3 University of Galway, Galway, Ireland University of Galway Galway Ireland; 4 University of the Basque Country (UPV/EHU), Leioa, Spain University of the Basque Country (UPV/EHU) Leioa Spain; 5 Plentzia Marine Station (PiE-UPV/EHU), Plentzia, Spain Plentzia Marine Station (PiE-UPV/EHU) Plentzia Spain; 6 Institute of Marine Biology, Biotechnology and Aquaculture (IMBBC), Hellenic Centre for Marine Research (HCMR), Heraklion, Greece Institute of Marine Biology, Biotechnology and Aquaculture (IMBBC), Hellenic Centre for Marine Research (HCMR) Heraklion Greece; 7 Royal Botanic Garden Edinburgh, Edinburgh, United Kingdom Royal Botanic Garden Edinburgh Edinburgh United Kingdom; 8 Department of Biological, Geological and Environmental Science, University of Bologna, Ravenna, Italy Department of Biological, Geological and Environmental Science, University of Bologna Ravenna Italy; 9 Flanders Marine Institute (VLIZ), Oostende, Belgium Flanders Marine Institute (VLIZ) Oostende Belgium; 10 Department of Environment, Faculty of Environment, Ionian University, Zakynthos, Greece Department of Environment, Faculty of Environment, Ionian University Zakynthos Greece; 11 Centro de Investigación Mariña, Universidade de Vigo, Vigo, Spain Centro de Investigación Mariña, Universidade de Vigo Vigo Spain; 12 Tvärminne Zoological Station, University of Helsinki, Hanko, Finland Tvärminne Zoological Station, University of Helsinki Hanko Finland; 13 National Institute of Biology, Marine Biology Station Piran, Piran, Slovenia National Institute of Biology, Marine Biology Station Piran Piran Slovenia; 14 European Marine Biological Resource Centre (EMBRC), Paris, France European Marine Biological Resource Centre (EMBRC) Paris France; 15 Department of Ecoscience, Marine Diversity and Experimental Ecology, Aarhus university, Aarhus, Denmark Department of Ecoscience, Marine Diversity and Experimental Ecology, Aarhus university Aarhus Denmark; 16 University of Gothenburg, Gothenburg, Sweden University of Gothenburg Gothenburg Sweden

## Abstract

Continuing the international efforts of the ARMS Marine Biodiversity Observation Network (ARMS-MBON), we present data from the second sampling campaign, coming from 56 Autonomous Reef Monitoring Structures (ARMS) deployed in 2020 and 2021 along European coasts under the European Marine Omics Biodiversity Observation Network (EMO BON). The dataset includes information on sampling locations and conditions, sample archiving and quality reports of collected samples. Data and metadata are openly accessible and can be downloaded from the associated GitHub repository. Sequence data can be accessed via the European Nucleotide Archive (ENA) through the corresponding accession numbers. Images of ARMS plates are stored on PlutoF and can be downloaded through links provided in this paper. Sequence data were processed and explored with the PEMA pipeline, resulting in 17,194, 7,235 and 5,261 unique ASVs/OTUs for COI, 18S and ITS, respectively. In this dataset, ARMS revealed the presence of over 61 eukaryotic phyla, aligning with our previous sampling campaign. Amongst these phyla, 35 had sequences identified to the species level. With this dataset and its associated paper, we provide a standardised resource for marine biodiversity monitoring and scientific analyses of benthic biodiversity. The presented data product supports future studies on the status and changes in species composition, distribution and genetic diversity.

## Introduction

In an era of significant global changes, the study and conservation of biodiversity have become a major focus for scientists worldwide. Only recently have the intricate connections between the health of these ecosystems and human well-being started to gain attention in public health discourse and decision-making, in the light of the One Health concept (Horton et al. 2014). Oceans, historically overlooked in conservation efforts, cover over 90% of the biosphere and are critical habitats teeming with wildlife (Cowan 1997). To this date, more than 240,000 marine species have been described (Ahyong et al. 2024) and an average of 2,332 new species are discovered every year (Bouchet et al. 2023). However, the distribution and ecology of most known species in the global ocean is still, to a large extent, unknown (Guidi et al. 2020), while data describing the population size and distribution range are lacking for the majority of marine species (Mora et al. 2011, Abreu et al. 2022. Investigating species distribution, range, abundance and genetic diversity is, therefore, a critical field of action for the conservation of marine systems.

However, most marine habitats face increasing threats, such as biodiversity loss and degradation (Mazaris et al. 2019, Luypaert et al. 2020, O’Hara et al. 2021), while processes, such as climate change, can significantly and abruptly alter the community composition and food web structure. Coastal areas are particularly vulnerable due to their proximity to human activities, for instance, tourism and industrial development (Harris et al. 2022, Green et al. 2022, González Hernández et al. 2023) and their sensitivity to climate change (Burkett et al. 2009). Understanding the dynamics between human activities and ecosystem response is more important than ever. To this end, the frameworks of Essential Biodiversity Variables (EBVs; Geijzendorffer et al. 2016, Proença et al. 2017, Kissling et al. 2018a, Kissling et al. 2018b), Essential Ocean Variables (EOVs; Miloslavich et al. (2018), Muller-Karger et al. (2018)), Essential Climate Variables (ECVs; Bojinski et al. (2014), Zeng et al. (2019)) and Essential Ecosystem Service Variables (EESVs; Balvanera et al. (2022)) have emerged to provide key metrics for monitoring biodiversity and oceanic changes at regional and global scales. EBVs aim to capture critical aspects of biodiversity change, such as species populations and genetic diversity, while EOVs focus on oceanic processes and ecosystem health. Given the complexity of marine environments and the often subtle nature of their ecological shifts, these frameworks highlight the necessity for innovative monitoring techniques and widely adhered protocols capable of delivering high-resolution insights into biodiversity patterns and changes. These techniques must operate on large scales and deliver high-resolution data to effectively capture the rapid loss of biodiversity and support conservation efforts, research and policy. Genetic methods, such as DNA/eDNA metabarcoding, have been developed and are increasingly used as they become cheaper and more efficient, while providing high taxonomic resolution (Taberlet et al. 2012, Ruppert et al. 2019). Moreover, consensus standardisation and public protocols have to be applied so results can be comparable across time and space. However, it is important to note that standardisation efforts for DNA/eDNA metabarcoding are still ongoing, with methodological choices (such as marker selection, primer design, sampling strategy and bioinformatic processing) continuing to influence results and comparability. Recent reviews and pilot studies emphasise the need for harmonisation of protocols and highlight current challenges and knowledge gaps that must be addressed for widespread adoption in marine biodiversity monitoring (van der Loos and Nijland 2021, Gold et al. 2022).

The Autonomous Reef Monitoring Structures Marine Biodiversity Observation Network (ARMS-MBON), initiated in 2018, carries out continuous genetic monitoring of hard-bottom communities across Europe (Obst et al. 2020) using standardised protocols (Suppl. material 1). Following the initial conceptual outlines by Obst et al. (2020) and Santi et al. (2023), the data from the first sampling campaign (2018-2020) were published in Daraghmeh et al. (2025). The occurrence data from this first dataset are available on the Global Biodiversity Information Facility (GBIF), the Ocean Biodiversity Information System (OBIS, EurOBIS) following the links in Suppl. material 2. These first years offered valuable perspectives on the pan-European genetic diversity of hard-bottom benthic ecosystems, demonstrated the effectiveness of DNA metabarcoding in improving traditional monitoring techniques and highlighted the obstacles faced in establishing a standardised marine monitoring network.

ARMS-MBON began under the ASSEMBLE Plus project (2017-2022) and is now coordinated by the European Marine Biological Resources Centre (EMBRC) under the European Marine Omics Biodiversity Observation Network (EMO BON) programme, led by EMBRC (Santi et al. 2023). EMO BON collects samples from the water column (*Wa*), soft substrates (*So*) and hard substrates using ARMS (*Ha*), aiming to allow researchers to explore marine diversity across different habitats. Thereby, EMO BON actively contributes to the UN Decade of Ocean Science for Sustainable Development and the global Ocean Biomolecular Observing Network initiative, which is one of the actions endorsed by the Decade (OBON; Meyer et al. (2023)).

Building on the foundations laid by this initial ARMS-MBON sampling campaign, the current data paper presents the second ARMS-MBON data release, focusing on 56 ARMS deployed along European coasts from 2020 to 2021. The dataset includes material samples, metadata, images, sequence data, derived taxonomic observations and documentation, all adhering to FAIR principles (Tanhua et al. 2019). This dataset contributes to the EBV framework by providing high-resolution genetic data that support monitoring of genetic diversity, species occurrence and community composition. Through standardised sampling and open-access data, it enables long-term, spatially comparable assessments of benthic biodiversity, aligning with key EBV classes, such as Genetic Composition and Species Populations.

### Value of the dataset

The dataset presented in this paper holds significant value for both marine ecology research and marine conservation programmes. It provides standardised, high-resolution genetic data on benthic species across European coasts. The data reported here are comparable with the previous and subsequent datasets from EMO BON observatories and, thereby, allow for the monitoring of the status and changes of benthic biodiversity. This dataset enhances biodiversity monitoring by contributing to Essential Biodiversity Variables (EBVs) and providing data on species occurrence, alpha/beta diversity and genetic diversity, addressing knowledge gaps in marine species distribution and diversity through 56 ARMS deployments. It supports scientific research, conservation planning and global interoperability by integrating with initiatives like the Ocean Biodiversity Information System (OBIS), the Global Biodiversity Information Facility (GBIF), the European Marine Observation and Data Network (EMODnet) and the European Digital Twin of the Ocean (EU DTO), see Data Resources 7 to 9.

Ultimately, this dataset provides a critical resource for understanding and protecting marine biodiversity in an era of rapid environmental change, offering long-term value for biodiversity research, ecosystem management and global conservation efforts.

## Methods

.

### Sampling, ARMS processing and image data

The original observatory design, fieldwork methodologies and sample processing procedures as well as instructions for biobanking and data management have been described in Obst et al. (2020). During this second ARMS MBON campaign, fieldwork, image data collection and sample collection followed the same guidelines as for the first sampling campaign (Daraghmeh et al. 2025), mainly guided by the ARMS MBON Handbook v2.0 and the EMO BON Handbook v2.0 that can be accessed on the ARMS-MBON GitHub repository, the EMO BON website and the Ocean Best Practices platform (see Suppl. material 1 for all useful links).

### General description of the sampling campaign

The presented data are derived from 56 ARMS units, corresponding to 56 individual sampling events. These include 55 unique Unit IDs, as two sampling events occurred at the same ARMS location (BelgiumCoast_AJJCD78, one deployment in 2020 and one in 2021, see Suppl. material 3).

#### Geographic coverage

**Figure 1.**
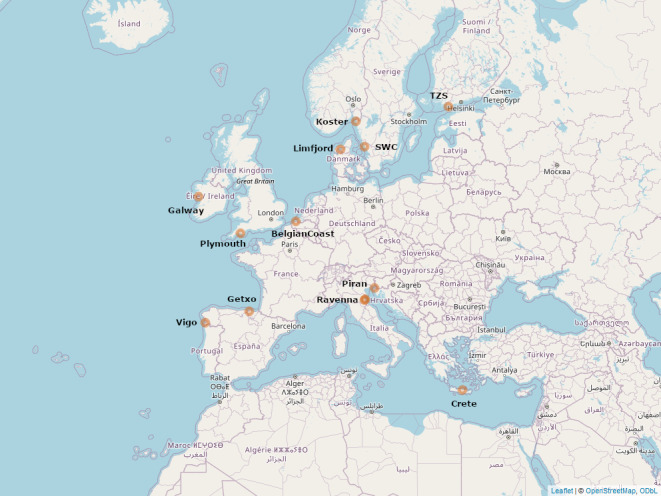
Locations of observatories that deployed ARMS units during the 2020–2021 ARMS-MBON sampling campaign. The two Ravenna (Italy) observatories are presented as a single entity here due to their proximity. TZS - Tvärminne Zoological Station. SWC - Swedish West Coast.

The dataset’s geographical range includes 13 observatories, across 10 countries, covering six ecoregions (Table 1, Fig. 1). For more information about the observatories and sites, see GitHub links in Suppl. material 1.

#### Temporal coverage

**Figure 2.**
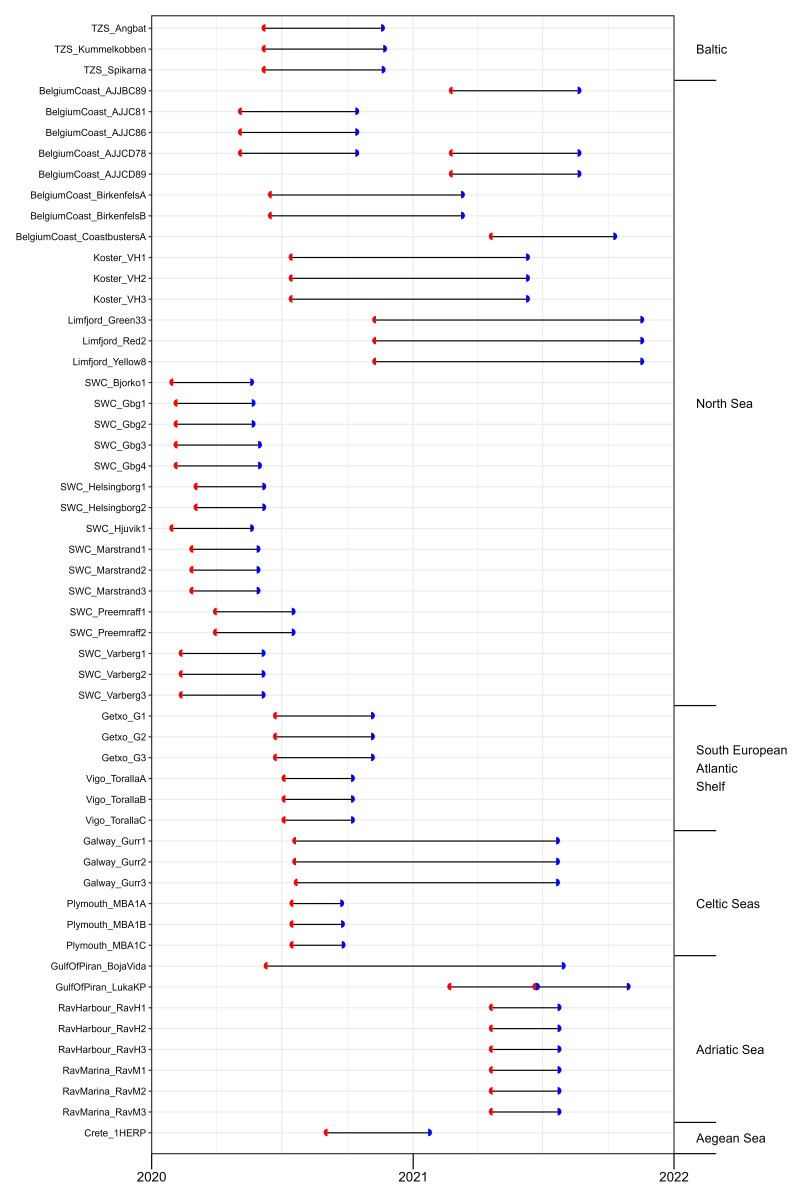
Sampling events of the second ARMS-MBON sampling campaign. Axis on the left shows ObservatoryID_UnitID combinations, axis on the right shows groupings of observatories into larger regions. Red semicircle: time of deployment. Blue semicircle: time of retrieval. Where red and blue semicircles meet, a new ARMS unit was deployed for a consecutive period at the same spot upon retrieval of the first unit. Where lines contain more than two semicircles (see GulfOfPiran_LukaKP), multiple units were deployed at the exact same spot at the same time, but were retrieved at different time points.

The dataset is composed of data from ARMS units deployed in 2020 and others in 2021 and retrieved during both years. These ARMS units were deployed during periods ranging between 66 days in Plymouth, UK and 412 days in Piran, Slovenia (Fig. 2). In fact, in earlier phases of ARMS MBON, different methodologies were tested (e.g. shorter deployments for alien species monitoring vs. longer deployments for long-term biodiversity monitoring). Additionally, longer deployments are needed at certain northern observatories (e.g. Svalbard) to ensure sufficient community development. Deployment time was also influenced by local constraints at the different partner stations. However, as more experience and information is gathered across the network, the goal is to standardise deployment duration in the future.

In the dataset presented by Daraghmeh et al. (2025), no significant linear association between deployment duration and the number of species identified or the ASV/OTU richness was detected. Deployment duration is, however, an important measure to track depending on which studies are performed with ARMS data.

General information on the observatories and ARMS deployments (e.g. coordinates, habitat type, deployment depth) and on the sampling events and their resulting material samples (e.g. date of deployment and retrieval, material sample IDs, preservative used) can be found on the GitHub repository (see Suppl. material 1 for respective links) and in Suppl. material 3.

### Laboratory protocols for amplicon sequencing

Until 2021, material samples had been sent to the Hellenic Centre for Marine Research (HCMR), Crete, Greece, for sequencing. This dataset is, therefore, composed of the three final sequencing batches processed in this facility, in September 2020, April 2021 and August 2023. The HCMR institute conducted amplicon sequencing for 162 material samples following the ARMS-MBON Molecular Standard Operational Procedure (see Suppl. material 1 for link). DNA metabarcoding was performed on the eukaryotic mitochondrial and nuclear marker genes cytochrome c oxidase subunit I (COI), 18S rRNA (18S) and for 68 samples, on the internal transcribed spacer (ITS) region. The COI primers primarily target metazoans, 18S amplifies a broad range of eukaryotes including protists and metazoans and ITS was used to better detect fungi and other microeukaryotes. The ITS marker was used exclusively during the project’s early stages and is currently no longer in use; this data paper, therefore, presents the last results for this marker.

For the COI marker gene, the *mlCOIintF* and *jgHCO2198* primers (Leray et al. 2013, Geller et al. 2013) were used and for 18S rRNA, *All18SF* and *All18SR* (Hardy et al. 2010) were chosen. Sequencing was performed with Illumina MiSeq following the ARMS MBON Molecular Standard Operating Procedures.

All raw sequence data from successful sequencing events are now publicly accessible in the European Nucleotide Archive (ENA) (see Data resources section below). Sequences from negative controls are also available. Additional details on how the sequence data were demultiplexed, including whether the reads include primer sequences, are documented in Suppl. material 4.

### Data management

Data management followed the same workflow as for our first data release, where the whole procedure has been described and documented (Daraghmeh et al. 2025). In short, event metadata (observatory, event and sample metadata, ENA accession numbers for sequencing data) were recorded on the ARMS-MBON project Googlesheet and ARMS plate images and associated spreadsheets were uploaded to the PlutoF platform (https://plutof.ut.ee/). After quality control (i.e. checking for inconsistent formats, missing entries or incomplete ones), all metadata and image data entered on PlutoF and all spreadsheet data were harvested and uploaded to the ARMS-MBON GitHub space (see Suppl. material 1 for links). When organising the data on GitHub, we created new repositories, specific to this second data release, but following the same structure as for the first (Daraghmeh et al. 2025). Each repository of the second data release was then packaged as a Research Object Crate (RO-Crate; Soiland-Reyes et al. (2022)) to produce machine-accessible and interoperable datasets. Raw sequence data are accessible on the European Nucleotide Archive via the accession numbers found on GitHub. The occurrence data obtained from the processing is integrated in the Global Biodiversity Information Facility (GBIF) and on the European Ocean Biodiversity Information System (OBIS), see Data Resources 7 to 9.

#### Biobanking

All partners were asked to keep at least one back-up replicate for each sample, stored in a freezer at -20°C, as well as a digital copy of all original images from the sampling event and the processed plates.

All collections of ARMS samples were carried out with the necessary ABS national permits. These allow the relevant stations to collect the samples in order to utilise the genetic material for taxonomic identification purposes. If anyone wishes to obtain and process any replicate material as stored in the individual partner stations, please note that any re-utilisation needs to be re-negotiated with ABS competent authorities in the providing countries.

### Data processing and exploration

#### Bioinformatics processing

A brief exploration of the sequencing outputs was performed to assess the quality and taxonomic coverage of the data. To this end, sequence data were processed using the Pipeline for Environmental DNA Metabarcoding Analysis, PEMA v.2.1.4 (Zafeiropoulos et al. 2020), as for the first data release. We used amplicon sequence variants (ASVs) for COI and ITS markers and operational taxonomic units (OTUs) for 18S, following PEMA guidelines. Taxonomy was assigned to 18S OTUs and ITS ASVs with the CREST LCAClassifier v.3.0 (Lanzén et al. 2012), using the PR2 v.4.13.0 (Guillou et al. 2013) and Unite v.7.2 (Nilsson et al. 2019) databases, respectively. For COI sequences, taxonomic annotation was performed using the RDP classifier (Wang et al. 2007) with the MIDORI database v.2.0 (Machida et al. 2017). For details on data processing, see Suppl. material 5, extracted from Daraghmeh et al. (2025), which describes the methods used.

The novelty of this second data release is that we ran PEMA using workflows developed by the European infrastructure LifeWatch ERIC (Arvanitidis et al. 2024). All PEMA runs were performed on the platform MyLifeWatch (https://my.lifewatch.eu/). On this platform, users can build workflows by connecting different wrappers (see Suppl. material 6 for additional information). We built our own workflow using: 1) the wrapper “PEMA Sequence Retriever” to obtain the raw data from ENA using the accession numbers for each sample (see Suppl. material 4); 2) the wrapper “Import File” to upload a parameter file and 3) the wrapper “PEMA Runner” to run PEMA using the outputs from the two previous wrappers. The workflows run in a Docker-based orchestrator that has a multi-server backend infrastructure currently established between datacentres from the Picasso Supercomputer (University of Malaga), the Scientific Computing Center of Andalusia (CICA, Seville), eBRIC (Huelva) and the University of Granada (Benítez-Hidalgo et al. 2021).

Data from the same sequencing batches were processed in the same workflow run, to allow the use of specific parameters for samples sequenced in similar conditions. All parameter files used for each PEMA run are available in the *analysis_release002* repository on the ARMS MBON GitHub (see Suppl. material 1 for respective link).

#### Blank correction and merge of the runs

We first performed a blank correction to discard any potential contaminant sequences, using the R package *decontam* v.1.20.0 (Davis et al. 2018) and the *prevalence* method. Results from *decontam* were double-checked prior removal of ASVs/OTUs (i.e. exact number of reads found in samples and in negative control, taxonomic classification of these potential contaminants). Sequences identified as contaminants by *decontam*, but that had many more reads in true samples than in negative controls and were classified in a marine taxa known in the study area, were kept in the dataset. Sequences identified as contaminants by *decontam* that had many more reads in the negative control or that were being classified as a contaminant taxa (e.g. Insecta, *Homo sapiens* etc.), were removed from the dataset. A list of the ASVs/OTUs that have been removed can be found in Suppl. material 7.

We then combined data from individual PEMA runs for each marker gene and curated the merged datasets to create a single dataset for visualisation and further inspection of the dataset. Since PEMA does not automatically apply a confidence threshold for taxonomic assignments of COI ASVs, we excluded all taxonomic assignments with confidence values below 0.8 for the rest of our analysis. Sequences with one of the following classifications were discarded as potential contaminants: *Homo sapiens*, *Canis lupus* or part of the following genera: *Bos*, *Felis*, *Sus*, *Gynaikothrips*, *Dorypteryx*, *Fannia*, *Bactrocera*, *Aleochara*, *Larus*, *Entomobrya*, *Hypogastrura*, *Psylla*, *Tanytarsus*, *Ptinus* and *Paratanytarsus*.

#### Taxonomic assessment

We analysed the data to determine the number and abundance of unique phyla, identified species and ASVs or OTUs classified to the species level, with ASVs used for COI and ITS markers and OTUs used for 18S. When reference databases failed to provide accurate phylum-level classification (see Bioinformatics processing), we manually corrected these classifications by consulting the World Register of Marine Species (WoRMS; Ahyong et al. (2024)) to identify the correct taxonomy. We then used custom code to update the classifications in the dataset, ensuring consistency and accuracy across all samples. We also checked for unique species found exclusively in any of the three markers datasets, as well as unique species detected by all of the three markers.

#### Occurrence data publication

Occurrence records generated in this study were formatted according to the Darwin Core (DwC) standard and compiled into DwC archives. The dataset was first deposited in the Integrated Marine Information System (IMIS, see Data Resources 7 to 9), from which the GBIF and OBIS publication links are accessible.

## Results

### Overall description

#### Genetic dataset

Out of the 56 retrieved ARMS, 162 material samples were obtained. Each ARMS unit was disassembled in the lab and its plates were carefully scraped and rinsed to collect the associated biological material. The collected material was then size-fractionated using sieves: typically into one sessile fraction (> 500 µm, organisms attached to plates) and two motile fractions (1000–500 µm and 500–100 µm). In most cases, all three fractions were processed per ARMS unit, but some sampling events yielded fewer fractions due to limited material or technical constraints. Of the 162 samples sequenced, 155 were successfully sequenced using both the COI and 18S marker genes (i.e. containing sequences deposited in ENA). For the ITS marker, 68 out of 69 processed samples were successfully sequenced (Table 2).

**Figure 3.**
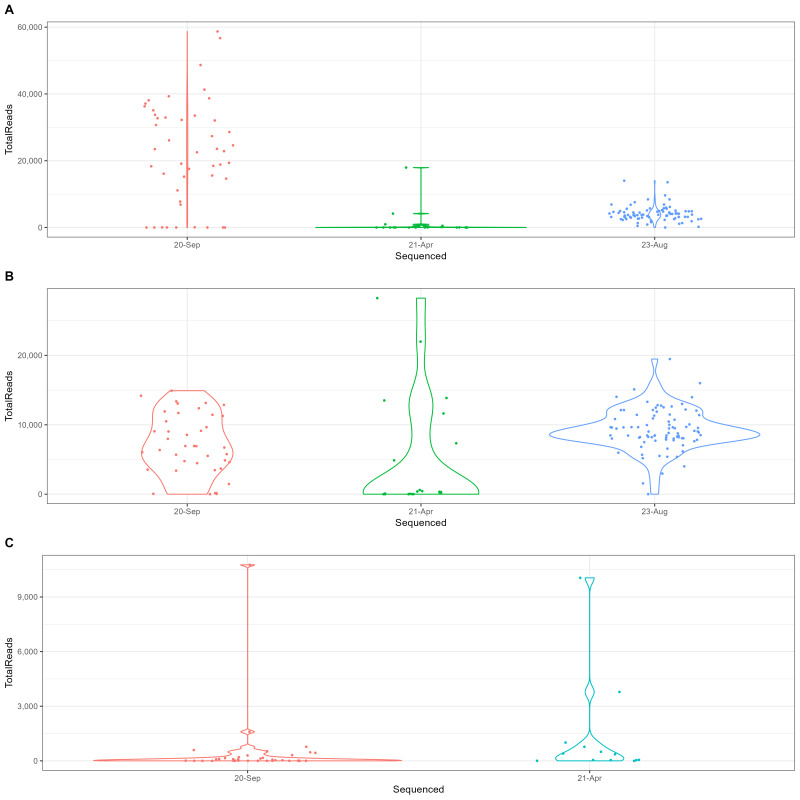
Sequencing depth of each sequencing batch (20-Sep, 21-Apr and 23-Aug), for COI (**A**), 18S (**B**) and ITS (**C**). The violin plots display the distribution of total reads for each sequencing batch, with the width of the plot indicating the density of reads at different depths.

We assessed the sequencing depth of each sequencing run. The distribution of total sequencing reads across different sequencing dates (20-Sep, 21-Apr and 23-Aug) and across markers (COI, 18S, ITS) showed notable variation (Fig. 3). We also compared the mean sequencing depth per sample of this dataset and of the previous one (Daraghmeh et al. 2025) for each marker (Suppl. material 8).

#### Image dataset

During this campaign, 8,362 photographs of ARMS plates were taken and stored on PlutoF. This comprises images of both sides of the settlement plates, as well as close-ups of individual specimens or colonies.

### Exploration of the genetic dataset

The sequenced data published on ENA and processed with PEMA, resulted in 17,194, 7,235 and 5,261 unique ASVs/OTUs for COI, 18S and ITS, respectively (Table 3). After further curation and filtering (i.e. negative-control-correction and removal of unclassified sequences), 153 samples with 17,175 ASVs remained for the COI dataset and 148 samples with 6,258 OTUs remained for the 18S dataset. For ITS, 50 samples with 645 ASVs remained (here, ASVs were inferred from only two sequencing runs, i.e. September 2020 and April 2021). This corresponded to 1,436,988 sequence reads for COI, 1,198,635 sequence reads for 18S and 34,105 sequence reads for ITS (Table 3). We also compared the average number of ASVs/OTUs per sample of this dataset and of the previous one (Daraghmeh et al. 2025) for each marker (Suppl. material 9) .

Combining the results from the three marker genes, 61 phyla have been recovered, including 30, 52 and 9 coming from the COI, 18S and ITS datasets, respectively. Amongst these 61 phyla, 35 have species-level identifications (Table 3), including 28, 27 and 15 from the COI, 18S and ITS datasets, respectively.

**Figure 4.**
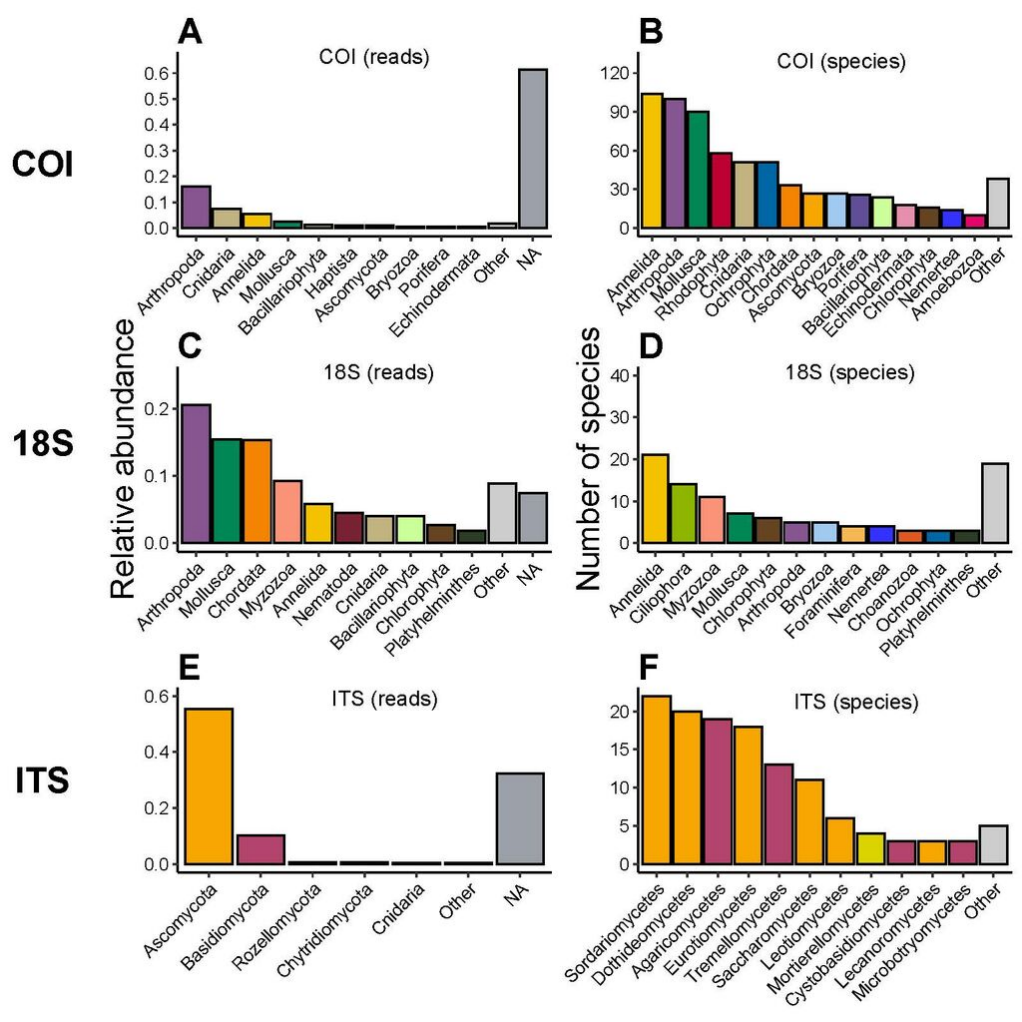
Relative read abundance of the ten most abundant phyla in the COI **(A)** and 18S **(C)** and five most abundant phyla in the ITS datasets **(E)**. Less abundant phyla are grouped as *Other*, while relative abundance of sequence reads unclassified at phylum level are grouped as *NA*. Number of identified species within each phylum for COI **(B)** and 18S **(D)** and within each class for ITS **(F)**. Phyla/classes with less than ten (i.e. COI) or three (i.e. 18S and ITS) identified species are grouped as *Other*. Colours correspond to the same unique phyla across all plots. Class level representation was chosen in **(F)** for better taxonomic resolution and colours correspond to the fungal phylum to which each class belongs.

When assessing the phyla’s relative read abundances for each marker, we found that the COI dataset is dominated by Arthropoda (16%), Cnidaria (7.5%), Annelida (5.5%) and Mollusca (2.5%), but also that more than half of the reads (61%) come from sequences that have not been classified at the phylum level (Fig. 4A). For 18S, the main phyla were Arthropoda (20%), Mollusca (15%), Chordata (15%) and Myzozoa (9%). Only around 7% of the reads could not be assigned to a phylum (Fig. 4C). In the case of ITS, the dataset was largely dominated by the phylum Ascomycota (55%), followed by Basidiomycota (10%). Here, 32% of the reads were not assigned to a phylum (Fig. 4E). Most of these common phyla were found across the whole study area (see Suppl. material 11). All species occurrences from data processing are published alongside this paper, see Data Resources 7 to 9.

**Figure 5.**
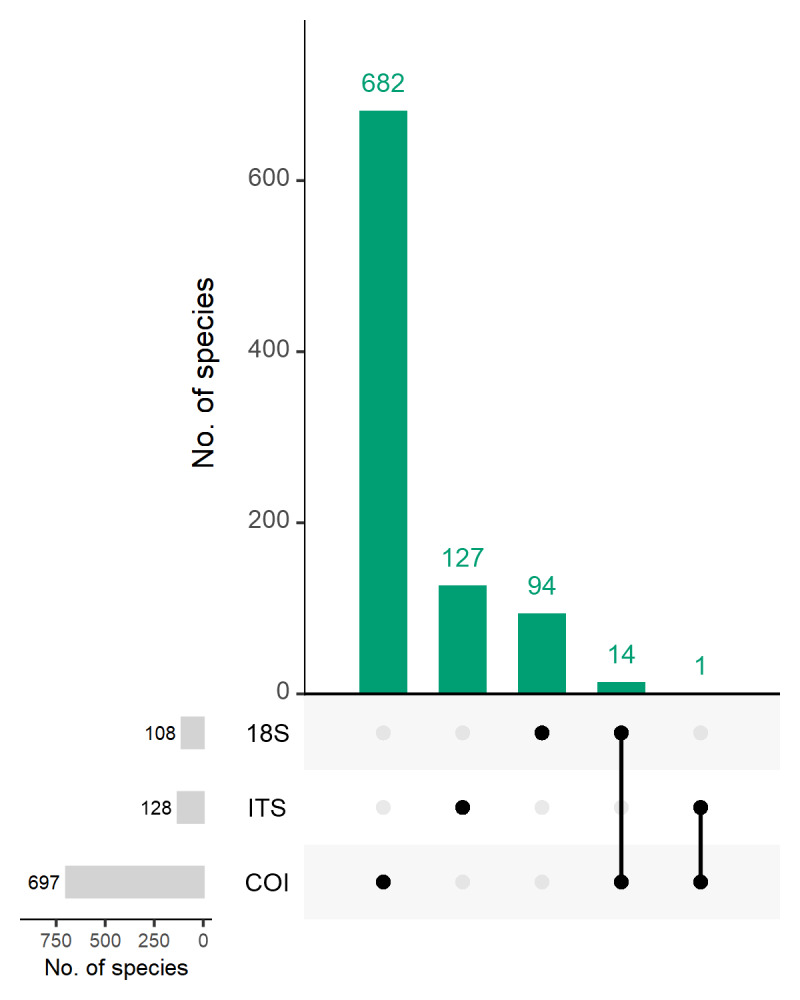
UpSet plot showing the number of species identified using the three marker genes: COI, 18S and ITS. Green bars represent the number of species identified (based on the applied confidence threshold) that are shared across the various combinations of marker gene datasets. The matrix below the bar plot indicates which combinations of marker genes correspond to each bar. Bars on the left display the total number of species identified within each marker gene dataset. Notably, no species were found to be common across all three datasets.

As expected, the three marker genes are complementary as they identify different groups of taxa (Fig. 4). This is supported by Fig. 5, which shows that 682 (out of 697), 94 (out of 108) and 127 (out of 128) identified species were unique to the COI, 18S and ITS datasets, respectively. Fourteen species were found in both the COI and 18S datasets, while only one was found across the ITS and COI datasets. No species were found in all three datasets.

At the genus level (Suppl. material 10), we observe a similar pattern: 491 (out of 523), 80 (out of 102) and 87 (out of 97) identified genera were unique to the COI, 18S and ITS datasets, respectively. Twenty-two genera were found in both the COI and 18S datasets, while ten were found across the ITS and COI datasets. No genera were found in all three datasets.

## Discussion

This paper presents the set of raw and processed amplicon sequencing data from the second sampling campaign of ARMS-MBON, from deployments performed in 2020 and 2021 and continues the genetic monitoring programme initiated in 2018. The raw sequencing data, image data and metadata are open-access (CC BY), allowing all interested users to use and reprocess them according to their specific needs. Additionally, we processed the sequencing data using PEMA v.2.1.4 (Zafeiropoulos et al. 2020) and all taxonomic occurrences will be accessible on our GitHub repositories, but also on public databases, such as GBIF and OBIS.

Ecological patterns observed in the dataset revealed distinct community compositions across markers and taxonomic groups. The COI dataset was dominated by Arthropoda, Cnidaria, Annelida and Mollusca, while 18S was largely composed of Arthropoda, Mollusca, Chordata and Myzozoa and ITS was heavily skewed towards Ascomycota and Basidiomycota (Fig. 4). These differences highlight the complementary nature of the marker genes and reinforce the need for a multi-marker approach in benthic biodiversity assessments. Furthermore, most species detected were unique to each marker, with minimal overlap, suggesting that each marker provides access to distinct portions of the community (Fig. 5). The data also confirmed the detection of 61 eukaryotic phyla, with 35 of them having ASVs/OTUs identified to species level, providing a broad taxonomic resolution that aligns with earlier findings in the network (Daraghmeh et al. 2025).

Raw data and the subsequent PEMA outputs presented here are comparable to our first data release (Daraghmeh et al. 2025) and to other similar studies (Leray and Knowlton 2015, Gielings et al. 2021, Coker et al. 2023). More specifically, for COI and ITS, the mean sequencing depth per sample was a similar range to that reported in Daraghmeh et al. (2025) (Suppl. material 8). However, it was lower for 18S, which could be due to lower sequencing quality. These patterns likely reflect differences in sample quality, sequencing conditions or library preparation across the dates and suggest potential batch effects that should be accounted for when performing further statistical analyses. Additionally, the average number of ASVs/OTUs per sample was 60% lower in the second release for COI, 17% lower for 18S and the same for ITS (Suppl. material 9). The number of species detected, however, was similar between both datasets for 18S (Wilcoxon, p = 0.68) and COI (Wilcoxon, p = 0.94). Differences between datasets can be due to different sequencing quality or differences in sample preparation protocols, sequencing platforms or bioinformatics processing pipelines (Smith and Peay 2014, Sims et al. 2014), which, however, has not been the case in this study. Variability in sequencing performance across markers may also reflect inherent differences in primer efficiency, target gene diversity or amplification success, all of which can influence the recovery of ASVs/OTUs. Multi-marker strategies have been recommended to improve taxonomic coverage (Portas et al. 2022) and provide more accurate community composition estimates (Günther et al. 2018, Stefanni et al. 2018).

The ARMS-MBON monitoring network has undergone significant growth and development since its initial years, marked by the addition of new observatories and an increasingly structured approach to sample collection, data management and analysis. This expansion has greatly enriched our dataset, enhancing our capacity for long-term monitoring of marine ecosystems. The integration into the EMO BON network in 2021, which conducts extensive water and sediment sampling across Europe, represents a major advancement.

Moreover, our ongoing efforts to refine methodologies are supported by the collective expertise and insights from the partners’ fieldwork and data analysis experiences. This continuous improvement cycle not only strengthens the monitoring capabilities of ARMS-MBON, but also ensures that our methods remain at the forefront of marine biodiversity research. The synergy between ARMS-MBON and EMO BON, alongside our commitment to methodological advancements, allow us to make substantial contributions to the understanding and preservation of marine ecosystems.

The ARMS-MBON network is well-equipped to provide comprehensive data for studying biodiversity patterns in marine ecosystems over the long term. For instance, it has already been shown that ARMS-BON network data are useful for the identification of NIS (Daraghmeh et al. 2025, Pagnier et al. 2025). Using a variety of methodologies, ARMS-MBON can generate detailed insights into species distribution, community structure and genetic diversity. The network’s extensive array of observatories facilitates the collection of high-resolution, spatially-diverse data, which is critical for understanding the complex dynamics of marine biodiversity. Additionally, the data generated align closely with Essential Biodiversity Variables (EBVs), ensuring compatibility with global biodiversity monitoring frameworks and enhancing the utility of the dataset for large-scale ecological assessments. This robust and expanding dataset enables rigorous statistical analyses, providing valuable information on both short-term patterns and long-term trends.

In addition to the extensive genetic data provided in this second data release, we also include a comprehensive set of high-resolution images from the deployed ARMS units. While these images remain underutilised in current analyses, they offer significant potential for complementary research. In fact, studies showed that ARMS photo-analyses can be used to compare marine benthic communities (David et al. 2019). Visual data can provide crucial context for the genetic findings by documenting the physical appearance of the communities, offering insights into species behaviour, habitat structures and possible environmental changes over time. Furthermore, the images could be used for cross-referencing with genetic data to improve species identification and validate the presence of non-indigenous species (NIS) detected via metabarcoding (Blair et al. 2024). This visual documentation also holds value for the development of more integrative approaches to biodiversity monitoring that combine genetic, ecological and visual data. We encourage the broader scientific community to explore the rich potential of this image dataset in future research.

## Benefits sharing statement

ARMS-MBON and EMO BON represent a large-scale research collaboration with scientists from across Europe and beyond. All network partners of the observatories mentioned in this manuscript provided genetic samples and are included as co-authors. All continuously generated raw and processed data from this network are shared with the public and scientific community (see above). Our research addresses the urgent need for large-scale and long-term monitoring of marine biotic communities through extensive collaborative efforts.

## Conflicts of interest

The authors declare that the research was conducted in the absence of any commercial or financial relationships that could be construed as a potential conflict of interest.

## Data Resources

Data and metadata are accessible through the following:

- Resource 1: Genetic data

see below

- Resource 2: Sample metadata:

https://github.com/arms-mbon/data_release_002

- Resource 3: Bioinformatics data and metadata (inputs, outputs, parameters):

https://github.com/arms-mbon/analysis_release_002

- Resource 4: Code used for the post-PEMA analyses:

https://github.com/arms-mbon/code_release_002

- Resource 5: Sample access (For physical samples, requests can be directed to the corresponding author or the institutions responsible for sample archiving, as detailed in the dataset documentation: All partners were asked to keep at least one back-up replicate for each sample, stored in a freezer at -20°C, as well as a digital copy of all original images from the sampling event and the processed plates):

https://www.embrc.eu

- Resource 6: Image data (Images of ARMS plates from this data set are stored on PlutoF and can be downloaded using the links provided in the dedicated CSV file below):

https://github.com/arms-mbon/data_release_002/blob/main/ImageData_release002.csv

- Resource 7: IMIS record for COI (where Darwin Core Archive files can be downloaded, links to GBIF, OBIS and EurOBIS records can be found):

https://www.vliz.be/en/imis?module=dataset&dasid=8922

- Resource 8: IMIS record for 18S (where Darwin Core Archive files can be downloaded, links to GBIF, OBIS and EurOBIS records can be found):

https://www.vliz.be/en/imis?module=dataset&dasid=8918

- Resource 9: IMIS record for ITS (where Darwin Core Archive files can be downloaded, links to GBIF, OBIS and EurOBIS records can be found):

https://www.vliz.be/en/imis?module=dataset&dasid=8921

### Resource 1

Download URL: All the raw sequence files and negative controls of this study were submitted to the European Nucleotide Archive (ENA) with the umbrella study accession number PRJEB72316 (publicly available at http://www.ebi.ac.uk/ena/data/view/PRJEB72316).

Resource identifier: The accession numbers of the component projects under the umbrella study are PRJEB37740, PRJEB37757, PRJEB33796, PRJEB37754, PRJEB37756, PRJEB37757, PRJEB72186, PRJEB37740, PRJEB37741, PRJEB37753, PRJEB72187, PRJEB37756, PRJEB37754 and PRJEB37744.

Data format : FASTQ

## Conclusion

In conclusion, the second data release of ARMS-MBON provides an enriched and continued dataset for benthic biodiversity in Europe, including both raw and processed amplicon sequencing data alongside high-resolution images. This release highlights the network’s commitment to advancing marine biodiversity monitoring through rigorous methodologies and integration with global frameworks, such as EBVs. By fostering accessibility, interoperability and sustainability, ARMS-MBON continues to make significant contributions to understanding and preserving marine ecosystems.

Conclusion

## Usage Rights

CC BY 4.0

Usage Rights

## Data accessibility statement

All data presented in this manuscript are publicly available with a CC BY licence (see main text and Supplementary Material for detailed descriptions). Standard operating procedures and protocols are available on the dedicated ARMS-MBON GitHub repository (https://github.com/arms-mbon/documentation). All metadata and access to all image data generated during this sampling campaign of ARMS-MBON to date can be found on GitHub (https://github.com/arms-mbon/data_release_002). All genetic raw data generated by ARMS-MBON to date can be accessed on the European Nucleotide Archive (ENA) through the accession numbers provided via the GitHub repository (https://github.com/arms-mbon/data_release_002) and under the umbrella study PRJEB72316 (https://www.ebi.ac.uk/ena/browser/view/PRJEB72316). Metadata, access to image data and accession numbers for genetic data specifically for the dataset presented in this manuscript are provided in the Supplementary Files and are also available on the respective GitHub repository (https://github.com/arms-mbon/data_release_002). All PEMA-processed data are accessible through the GitHub repository (https://github.com/arms-mbon/analysis_release_002) and occurrences data will be accessible via GBIF and OBIS.

Data accessibility statement

## Supplementary Material

C8D7D125-6C0E-5E2F-889D-252CDD8DDB96 10.3897/BDJ.13.e148981.suppl1 Supplementary material 1 Supplementary Table S1 Data type table Brief description Overview of ARMS-MBON and EMO BON project web pages, GitHub repositories and IMIS metadata records for taxonomic occurrences of data release 002 submitted to EurOBIS. File: oo_1354362.docx https://binary.pensoft.net/file/1354362 Pagnier et al.

9CA7318B-A10D-5DF6-8FC7-4DEA8C136019 10.3897/BDJ.13.e148981.suppl2 Supplementary material 2 Supplementary Table S2 Data type table Brief description Table summarising links to GBIF, OBIS and EurOBIS datasets from the first data release of ARMS MBON (Daraghmeh et al. 2025). File: oo_1354358.docx https://binary.pensoft.net/file/1354358 Pagnier et al. (2025)

715F9DDB-3D31-5176-9E62-2BEC2C60544D 10.3897/BDJ.13.e148981.suppl3 Supplementary material 3 Sample metadata Data type csv File: oo_1355167.csv https://binary.pensoft.net/file/1355167 Pagnier et al.

1D2EA5D2-E2BF-55D5-898D-FACFD4935710 10.3897/BDJ.13.e148981.suppl4 Supplementary material 4 List of ENA numbers Data type csv File: oo_1216700.csv https://binary.pensoft.net/file/1216700 Pagnier et al.

6A620D54-4DA7-5A7C-B98B-0FC59CC8C224 10.3897/BDJ.13.e148981.suppl5 Supplementary material 5 Supplementary Text S1 Data type text Brief description Bioinformatics processing of raw sequence data (extracted from Daraghmeh et al. (2024), first data release). File: oo_1354353.docx https://binary.pensoft.net/file/1354353 Pagnier et al.

C7441573-1B2A-5829-8CCD-B2F8910153C1 10.3897/BDJ.13.e148981.suppl6 Supplementary material 6 Supplementary Text S2 Data type text Brief description Description of the MyLifeWatch platform and Workflow Studio. File: oo_1354354.docx https://binary.pensoft.net/file/1354354 Pagnier et al.

B866429F-A9A2-55D8-BF2A-61C708E94584 10.3897/BDJ.13.e148981.suppl7 Supplementary material 7 Details of the blank correction Data type xlsx File: oo_1216706.xlsx https://binary.pensoft.net/file/1216706 Pagnier et al.

140312D7-6270-55B8-98FE-6E113F1C38C2 10.3897/BDJ.13.e148981.suppl8 Supplementary material 8 Supplementary Figure S1 Data type graph Brief description Bar plot showing the mean sequencing depth per sample for each marker in both Data Paper 1 (Daraghmeh et al. (2024)) and Data Paper 2 (this present data release). File: oo_1355171.docx https://binary.pensoft.net/file/1355171 Pagnier et al.

81213F33-9BE0-54EC-9A22-AFA6CDBC6E91 10.3897/BDJ.13.e148981.suppl9 Supplementary material 9 Supplementary Figure S2 Data type graph Brief description Bar plot showing the mean number of ASVs/OTUs per sample for each marker in both Data Paper 1 (Daraghmeh et al. (2024)) and Data Paper 2 (this present data release). File: oo_1216708.docx https://binary.pensoft.net/file/1216708 Pagnier et al.

5CFBCCFF-E422-50DC-83C6-8B1BEE30A6BD 10.3897/BDJ.13.e148981.suppl10 Supplementary material 10 Supplementary Figure S3 Data type graph Brief description UpSet plot showing the number of genera identified using the three marker genes: COI, 18S and ITS. File: oo_1216710.docx https://binary.pensoft.net/file/1216710 Pagnier et al.

F4F7F069-73D8-5651-9136-F6A58FF33197 10.3897/BDJ.13.e148981.suppl11 Supplementary material 11 Geographic distribution of the 10 most abundant phyla (for 18S and COI) Data type images Brief description Two maps in a Word file showing the geographic distribution of the 10 most abundant phyla (in terms of ASVs/OTUs) for COI and 18S. File: oo_1354209.docx https://binary.pensoft.net/file/1354209 Pagnier et al. (2025)

## Figures and Tables

**Table 1.** **Table 1**. Locality and geographical coordinates of the observatories collecting ARMS samples during the second sampling campaign. Ecoregions are according to Spalding et al. (2007).

Locality	Country	Coordinates	Ecoregion	Deployment dates (deployment -- retrieval)
RavennaM	Italy	44.492426N; 12.287623E	Adriatic Sea	(22/04/2021 -- 22/07/2021)
RavennaH	Italy	44.421488N; 12.209497E	Adriatic Sea	(22/04/2021 -- 22/07/2021)
Vigo	Spain	42.2284N; -8.7787W	South European Atlantic Shelf	(06/07/2020 -- 06/10/2020)
Crete	Crete	35.343153N; 25.136605E	Aegean Sea	(03/09/2020 -- 22/01/2021);
Plymouth	United Kingdom	50.3673N; -4.1554W	Celtic Seas	(17/07/2020 -- 21/09/2020); (17/07/2020 -- 21/09/2020); (17/07/2020 -- 23/09/2020)
PiEGetxo	Spain	43.33858N; -3.014639W	South European Atlantic Shelf	(24/06/2020 -- 03/11/2020);
Belgium Coast	Belgium	51.43333 N; 2.808331 E	North Sea	(06/05/2020 -- 12/10/2020); (25/02/2021 -- 19/08/2021); (17/06/2020 -- 09/03/2021); (22/04/2021 -- 08/10/2021)
Galway	Ireland	53.315443N; -9.671564W	Celtic Seas	(21/07/2020- 08/07/2021); (21/07/2020 -- 07/07/2021); (23/07/2020 -- 08/07/2021)
Piran	Slovenia	45.54875N; 13.5507E	Adriatic Sea	(11/06/2020 -- 28/07/2021); (22/06/2021 -- 27/10/2021); (23/02/2021 -- 22/06/2021)
TZS	Finland	59.841505N; 23.248879E	Baltic Sea	(08/06/2020 -- 17/11/2020); (08/06/2020 -- 18/11/2020); (08/06/2020 -- 20/11/2020);
Koster	Sweden	58.875155N; 11.103194E	North Sea	(16/07/2020 -- 08/06/2021)
SWC	Sweden	57.1107004N; 12.2439775E	North Sea	(31/01/2020 --18/05/2020); (06/02/2020 -- 20/05/2020); (06/02/2020 -- 29/05/2020); (05/03/2020 -- 04/06/2020); (31/01/2020 -- 18/05/2020); (28/02/2020 -- 27/05/2020); (01/04/2020 -- 15/07/2020); (13/02/2020 -- 03/06/2020)
Limfjord	Denmark	56.89985 N; 9.05663333 E	North Sea	(10/11/2020 -- 15/11/2021)

**Table 2.** **Table 2**. Overview of the processed samples from the second ARMS-MBON sampling campaign. * ITS amplicon sequencing has since been discontinued, these being the last sequencing batches for this marker.

Sample collection	Overview		
Number of ARMS units retrieved	56		
Number of derived material samples (i.e. biological samples and technical replicates)	162		
Photographic images obtained from ARMS units	8,362		
Marker gene sequencing	COI	18S	ITS
Sequencing batches	3	3	2
Number of samples sequenced successfully (i.e. number of deposited ENA accessions)	155	155	68
Number of negative controls available	3	3	2

**Table 3.** **Table 3.** Overview of results from the sequence data processing using PEMA.

	**COI**	**18S**	**ITS**
Overall number of unique ASVs/OTUs prior to any curation	17,194	7,235	5,261
Number of PEMA-processed samples with classified ASVs/OTUs remaining after negative control correction (excl. negative controls)	153	148	50
Number of unique, classified, blank-corrected ASVs/OTUs	17,175	6,258	645
Number of ASVs/OTUs after curation and filtering	17,161	6,251	642
Sequencing depth (total read number of unique, classified, negative-control-corrected ASVs/OTUs)	1,436,988	1,198,635	34,105
Number of phyla recovered	30	52	9
ASV/OTUs with Linnean species name classification (with the confidence thresholds applied here)	1,502	193	138
Number of unique species identified with Linnean name	697	108	128
Number of phyla represented in species identifications	28	27	3 (15 classes)
	**Combined**		
Number of phyla recovered	61		
Number of phyla represented in species identifications	35		
