# Supplementary material for: A long-term ecological research dataset from the marine genetic monitoring programme ARMS-MBON 2020-2021
Source: Biodivers Data J. 2025 Nov 21;13:e148981. doi: 10.3897/BDJ.13.e148981 (PMC12663723; doi:10.3897/BDJ.13.e148981)
Supplement: Supplementary material 1 — Supplementary Table S1 [file bdj-13-e148981-s001.docx]

Supplementary Information to

A long-term ecological research data set from the genetic monitoring program ARMS- MBON 2020-2021

Corresponding author: Matthias Obst, Department of Marine Sciences, University of Gothenburg, matthias.obst@marine.gu.se

**Supplementary Table S2.** Overview of ARMS-MBON and EMO BON project web pages, GitHub repositories and IMIS metadata records for taxonomic occurrences of data release 002 submitted to EurOBIS.

| **ARMS-MBON main webpages and GitHub documentation and data repositories** |
| --- |
| *ARMS-MBON data landing page*  <https://data.arms-mbon.org> |
| *ARMS-MBON main GitHub page*  <https://github.com/arms-mbon> |
| *Documentation repository*  <https://github.com/arms-mbon/documentation> |
| *ARMS MBON handbook (prior to EMO BON time)* |
| <https://github.com/arms-mbon/documentation/tree/main/armsmbon_handbook/old> |
| *Handbook applied for ARMS in EMO BON*  <https://github.com/arms-mbon/documentation/tree/main/armsmbon_handbook> |
| *EMO BON handbook v2.0* |
| [*https://www.embrc.eu/services/emo-bon*](https://www.embrc.eu/services/emo-bon) |
| [*https://repository.oceanbestpractices.org/handle/11329/1738*](https://repository.oceanbestpractices.org/handle/11329/1738) |
| *Molecular Standard Operating Procedures (MSOP)*  <https://github.com/arms-mbon/documentation/tree/main/standard_operating_procedures> |
| *All ARMS-MBON harvested metadata and analysis data organized in folders*  <https://github.com/arms-mbon/data_workspace> |
| *All ARMS-MBON quality-controlled observatory, sampling event, image and genetic metadata*  <https://github.com/arms-mbon/data_workspace/tree/main/qualitycontrolled_data/combined> |
| ***data_release_002* repository** |
| *data_release_002 main page*  [*https://github.com/arms-mbon/data_release_002*](https://github.com/arms-mbon/data_release_002) |
| *Info on observatories for which data was analysed for this data release*  [*https://github.com/arms-mbon/data_release_002/blob/main/ObservatoryData_release002.csv*](https://github.com/arms-mbon/data_release_002/blob/main/ObservatoryData_release002.csv) |
| *Info on sampling events and material samples*  [*https://github.com/arms-mbon/data_release_002/blob/main/SamplingEventData_release002.csv*](https://github.com/arms-mbon/data_release_002/blob/main/SamplingEventData_release002.csv) |
| *Download links for ARMS image data*  [*https://github.com/arms-mbon/data_release_002/blob/main/ImageData_release002.csv*](https://github.com/arms-mbon/data_release_002/blob/main/ImageData_release002.csv) |
| *Info on amplicon sequencing data and corresponding ENA accession numbers*  [*https://github.com/arms-mbon/data_release_002/blob/main/OmicsData_release002.csv*](https://github.com/arms-mbon/data_release_002/blob/main/OmicsData_release002.csv) |
| ***analysis_release_002* repository** |
| *analysis_release_002 main pain*  [*https://github.com/arms-mbon/analysis_release_002/tree/main*](https://github.com/arms-mbon/analysis_release_002/tree/main) |
| *Curated taxonomy and occurrences*  [*https://github.com/arms-mbon/analysis_release_002/tree/main/taxonomic_assignments*](https://github.com/arms-mbon/analysis_release_002/tree/main/taxonomic_assignments) |
| *PEMA parameters used during data processing*  [*https://github.com/arms-mbon/analysis_release_002/tree/main/parameter_files*](https://github.com/arms-mbon/analysis_release_002/tree/main/parameter_files) |
| *Fasta files post processing (via Marine Data Archive)*  [*https://github.com/arms-mbon/analysis_release_002/tree/main/fasta*](https://github.com/arms-mbon/analysis_release_002/tree/main/fasta) |
