# Supplementary material for: A long-term ecological research dataset from the marine genetic monitoring programme ARMS-MBON 2020-2021
Source: Biodivers Data J. 2025 Nov 21;13:e148981. doi: 10.3897/BDJ.13.e148981 (PMC12663723; doi:10.3897/BDJ.13.e148981)
Supplement: Supplementary material 2 — Supplementary Table S2 [file bdj-13-e148981-s002.docx]

Supplementary Information to

A long-term ecological research data set from the genetic monitoring program ARMS- MBON 2020-2021

Corresponding author: Matthias Obst, Department of Marine Sciences, University of Gothenburg, matthias.obst@marine.gu.se

**Supplementary Table S1. Links to the dataset of the first release from ARMS MBON on GBIF, OBIS and EurOBIS.**

| **GBIF** |
| --- |
| *COI →* <https://www.gbif.org/dataset/b9afe2d0-b264-4422-bf8c-096b2a53c18f> |
| *18S →* [*https://www.gbif.org/dataset/6f2f07f3-1ef4-4f82-b3d4-d3bd1406650f*](https://www.gbif.org/dataset/6f2f07f3-1ef4-4f82-b3d4-d3bd1406650f) |
| *ITS →* <https://www.gbif.org/dataset/c6cbf3f2-9bfe-489d-9d33-226fe236275b> |
|  |
| **OBIS** |
| *COI →*<https://obis.org/dataset/066f002f-58d5-4687-bdb8-b39cdaef0c2b> |
| *18S →*<https://obis.org/dataset/0ada9b0c-14f5-4247-881e-9f6f62b2c165> |
| *ITS →* [*https://obis.org/dataset/ddab58b2-0072-41b8-afc5-ac10d937247f*](https://obis.org/dataset/ddab58b2-0072-41b8-afc5-ac10d937247f) |
|  |
| **EurOBIS** |
| *COI →* [https://www.eurobis.org/imis?module=dataset&dasid=8357](https://obis.org/dataset/066f002f-58d5-4687-bdb8-b39cdaef0c2b) |
| *18S →*<https://www.eurobis.org/imis?module=dataset&dasid=8617> |
| *ITS →* [*https://www.eurobis.org/imis?module=dataset&dasid=8612*](https://www.eurobis.org/imis?module=dataset&dasid=8612) |
