# Supplementary material for: A long-term ecological research dataset from the marine genetic monitoring programme ARMS-MBON 2020-2021
Source: Biodivers Data J. 2025 Nov 21;13:e148981. doi: 10.3897/BDJ.13.e148981 (PMC12663723; doi:10.3897/BDJ.13.e148981)
Supplement: Supplementary material 5 — Supplementary Text S1 [file bdj-13-e148981-s005.docx]

Supplementary Information to

A long-term ecological research data set from the genetic monitoring program ARMS- MBON 2020-2021

Corresponding author: Matthias Obst, Department of Marine Sciences, University of Gothenburg, matthias.obst@marine.gu.se

**Supplementary Text S1: Bioinformatics processing of raw sequence data (extracted from Daraghmeh et al. 2024, first data release)**

Sequence data were processed with the Pipeline for Environmental DNA Metabarcoding Analysis, PEMA v.2.1.4 (Zafeiropoulos et al., 2020). PEMA currently supports the analysis of five marker genes (12S, 16S, 18S rRNA, COI, and ITS), and may also be used for other genes of interest if a taxonomic reference database is provided. PEMA consists of four main steps: (i) sequence pre-processing, (ii) OTU clustering or ASV inference, (iii) taxonomic assignment, and (iv) optionally the performance of biodiversity analysis based on the taxonomic inventory retrieved. It has been shown that parameter settings can lead to rather different outcomes (Brandt et al., 2021; Zafeiropoulos et al., 2020). Therefore, for comparison reasons, a fixed set of parameters was used for each marker gene and sequence data were processed separately for each sequencing run. All parameter files with their specific settings used as input for PEMA runs are available on the ARMS-MBON GitHub repository (see Supplementary Table S1 for respective link). For the 18S marker gene, sequences were clustered into operational taxonomic units (OTUs) using the VSEARCH v2.9.1 algorithm (Rognes et al., 2016) with a threshold of 0.97, while for ITS and COI, clustering was performed with Swarm v2 (Mahe et al., 2015), applying a threshold of d = 10 to infer amplicon sequence variants (ASVs). Note that PEMA initially defined the result of Swarm processing as inferred ASVs, i.e., sequences which differ by one or more nucleotides, which is now corrected to swarm-clusters (Hakimzadeh et al., 2023). We here use PEMA’s terminology for consistency reasons. Taxonomy was assigned to 18S OTUs and ITS ASVs with the CREST LCAClassifier v3.0 (Lanzè et al., 2012), using the PR2 v.4.13.0 (Guillou et al., 2013) and Unite v7.2 (Nilsson et al., 2018) databases, respectively. Default settings were: i) minimum bit-score = 155; ii) LCA bitscore range = 2%; and iii) similarity cut-offs of 99%, 97%, 95%, 90%, 85% and 80% for the species, genus, family, order, class and phylum ranks, respectively. For COI sequences, taxonomic annotation was performed using the RDP classifier (Wang et al., 2007) with the MIDORI database v2.0 (Machida et al., 2017), and confidence values for each rank assignment were recorded, but no threshold was applied. Singletons (sequences with a total read abundance of one), OTUs/ASVs unclassified at domain level, and potential contaminant sequences (OTUs/ASVs that were more abundant in the negative control samples compared to actual samples) were removed. For OTUs/ASVs that were present in negative control samples in lower abundances than in actual samples, their corresponding read number in the negative controls was subtracted from their read number in actual samples. All bioinformatics analyses were supported by a distributed infrastructure in Spain, including data centers at Picasso (Malaga), CICA (Seville), eBRIC (Huelva), and the University of Granada.
