# Supplementary material for: A long-term ecological research dataset from the marine genetic monitoring programme ARMS-MBON 2020-2021
Source: Biodivers Data J. 2025 Nov 21;13:e148981. doi: 10.3897/BDJ.13.e148981 (PMC12663723; doi:10.3897/BDJ.13.e148981)
Supplement: Supplementary material 6 — Supplementary Text S2 [file bdj-13-e148981-s006.docx]

Supplementary Information to

A long-term ecological research data set from the genetic monitoring program ARMS- MBON 2020-2021

Corresponding author: Matthias Obst, Department of Marine Sciences, University of Gothenburg, [matthias.obst@marine.gu.se](mailto:matthias.obst@marine.gu.se)

**Supplementary Text S2: Description of the MyLifeWatch platform and Workflow Studio**

The MyLifeWatch platform, developed by LifeWatch ERIC, serves as an advanced infrastructure designed to support biodiversity research through enhanced access to datasets, tools, and virtual environments. A central feature of this platform is the Workflow Studio, which facilitates the design, modification, and execution of data analysis workflows, promoting a streamlined and efficient research process.

This feature supports the integration of various data inputs, analytical tools, and processing steps into a coherent workflow that can be saved, shared, and reused. Users can drag and drop different components (*i.e.* wrappers) and connect them to form a workflow that matches their project requirements. This includes data preprocessing, analysis, visualization, and post-processing steps.

This platform was chosen for our data processing because of the various advantages it has. By enabling the creation of explicit and reusable workflows, the studio enhances the reproducibility of research findings. Researchers can document their data flow, parameters, and processing steps in detail. Workflow Studio is seamlessly integrated with the broader MyLifeWatch ecosystem, allowing direct access to an extensive range of datasets and tools from within the platform. This integration ensures that workflows can utilize up-to-date and comprehensive data sources, such as GBIF, Zenodo, and other specialized databases.

By standardizing the analytical processes, the Workflow Studio helps in minimizing errors that might occur due to manual data handling or inconsistencies in analysis procedures. This standardization is vital for maintaining high data quality and reliability in biodiversity research. It also provides the flexibility to customize components based on their project's needs, allowing for a high degree of specificity in data analysis, which is often required in targeted biodiversity studies.

The Workflow Studio in the MyLifeWatch platform exemplifies LifeWatch ERIC’s commitment to providing advanced tools that enhance the efficiency, reproducibility, and collaborative potential of biodiversity research. By integrating comprehensive data access with powerful workflow management capabilities, the Workflow Studio stands out as a pivotal feature for researchers aiming to conduct innovative and impactful studies in the field of ecology and environmental science.
