# Supplementary material for: A long-term ecological research dataset from the marine genetic monitoring programme ARMS-MBON 2020-2021
Source: Biodivers Data J. 2025 Nov 21;13:e148981. doi: 10.3897/BDJ.13.e148981 (PMC12663723; doi:10.3897/BDJ.13.e148981)
Supplement: Supplementary material 8 — Supplementary Figure S1 [file bdj-13-e148981-s008.docx]

Supplementary Information to

A long-term ecological research data set from the genetic monitoring program ARMS- MBON 2020-2021

Corresponding author: Justine Pagnier, Department of Marine Sciences, University of Gothenburg, justine.pagnier@.gu.se


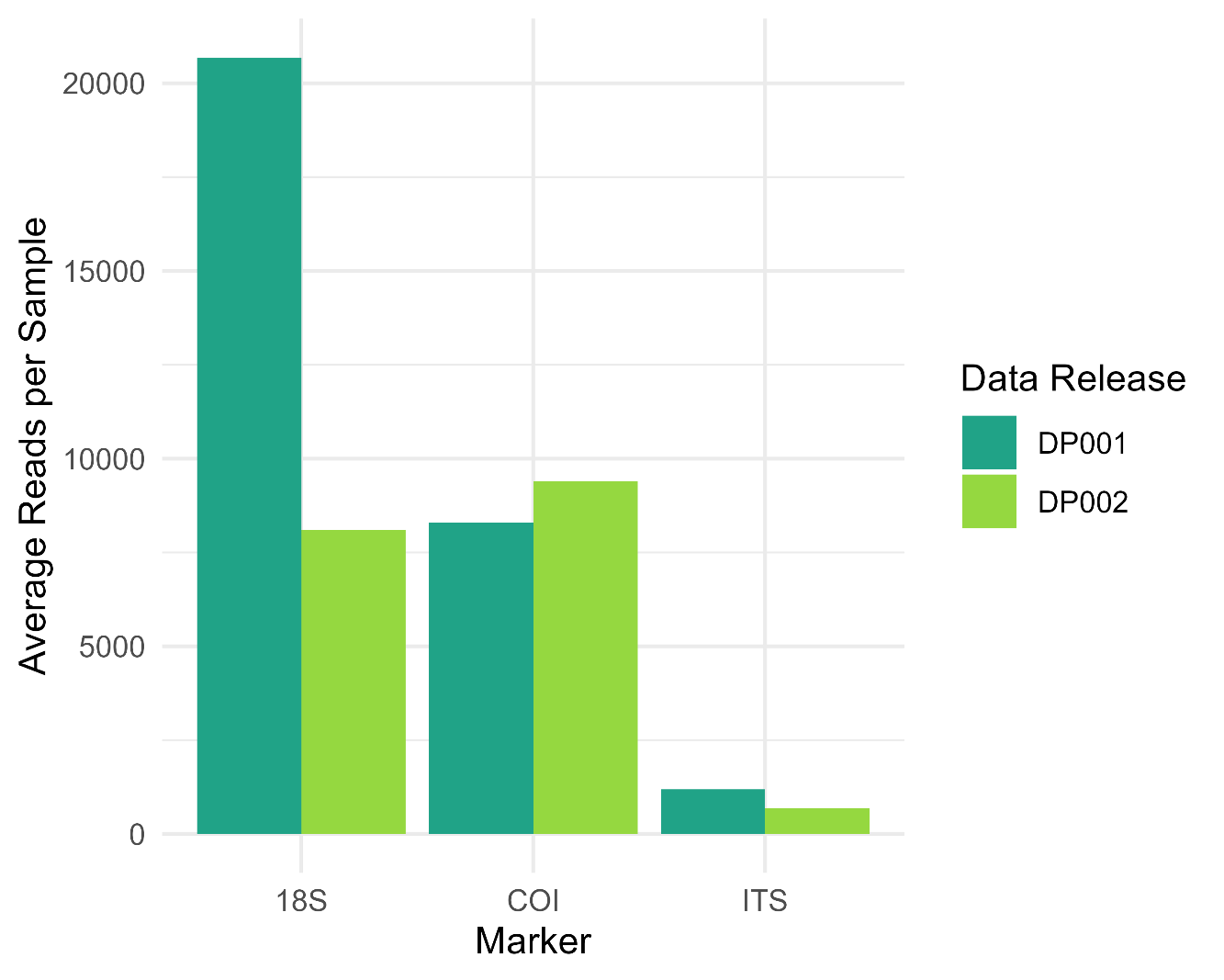


**Supplementary Figure S2.** Bar plot showing the mean number of reads per sample for each marker in both Data Paper 1 (dark green, Daraghmeh et al., 2024) and Data Paper 2 (light green, this present data release).
