# Supplementary material for: A long-term ecological research dataset from the marine genetic monitoring programme ARMS-MBON 2020-2021
Source: Biodivers Data J. 2025 Nov 21;13:e148981. doi: 10.3897/BDJ.13.e148981 (PMC12663723; doi:10.3897/BDJ.13.e148981)
Supplement: Supplementary material 10 — Supplementary Figure S3 [file bdj-13-e148981-s010.docx]

Supplementary Information to

A long-term ecological research data set from the genetic monitoring program ARMS- MBON 2020-2021

Corresponding author: Justine Pagnier, Department of Marine Sciences, University of Gothenburg, justine.pagnier@.gu.se


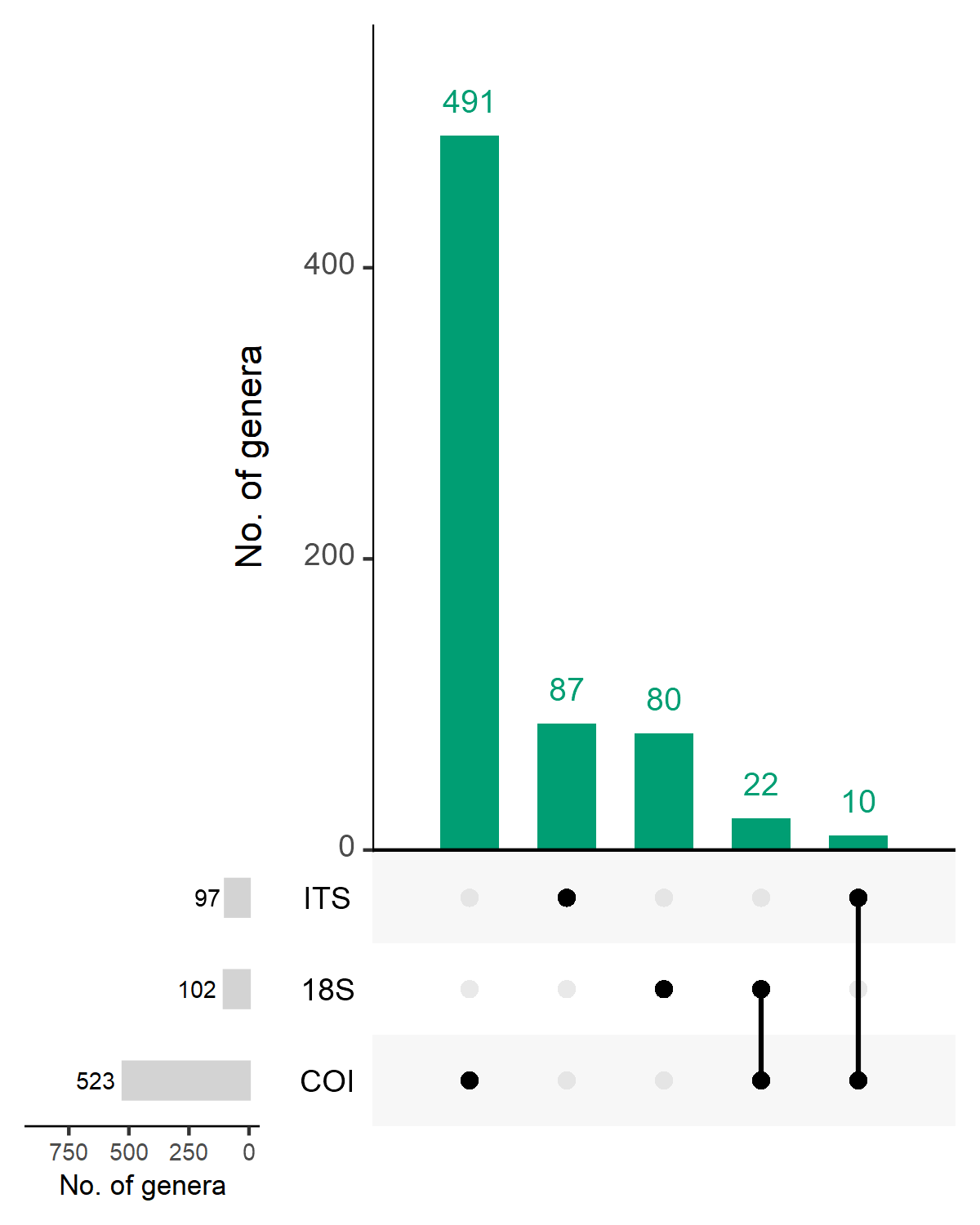


**Supplementary Figure S3.** UpSet plot showing the number of genera identified using the three marker genes: COI, 18S, and ITS. Green bars represent the number of genera identified that are shared across the various combinations of marker gene data sets. The matrix below the bar plot indicates which combinations of marker genes correspond to each bar. Bars on the left display the total number of genera identified within each marker gene data set. Notably, no genera were found to be common across all three data sets.
