# Supplementary material for: A long-term ecological research dataset from the marine genetic monitoring programme ARMS-MBON 2020-2021
Source: Biodivers Data J. 2025 Nov 21;13:e148981. doi: 10.3897/BDJ.13.e148981 (PMC12663723; doi:10.3897/BDJ.13.e148981)

Supplementary Information to

A long-term ecological research data set from the genetic monitoring program ARMS- MBON 2020-2021

Corresponding author: Justine Pagnier, Department of Marine Sciences, University of Gothenburg, justine.pagnier@.[gu.se](http://gu.se)

Supplementary Figures S4 to S5

**Supplementary Figure S4.** Geographic distribution of the 10 most abundant phyla (in terms of number of OTUs) detected with 18S marker. Differences in sampling effort, deployment duration or sequencing depth have not been accounted for.


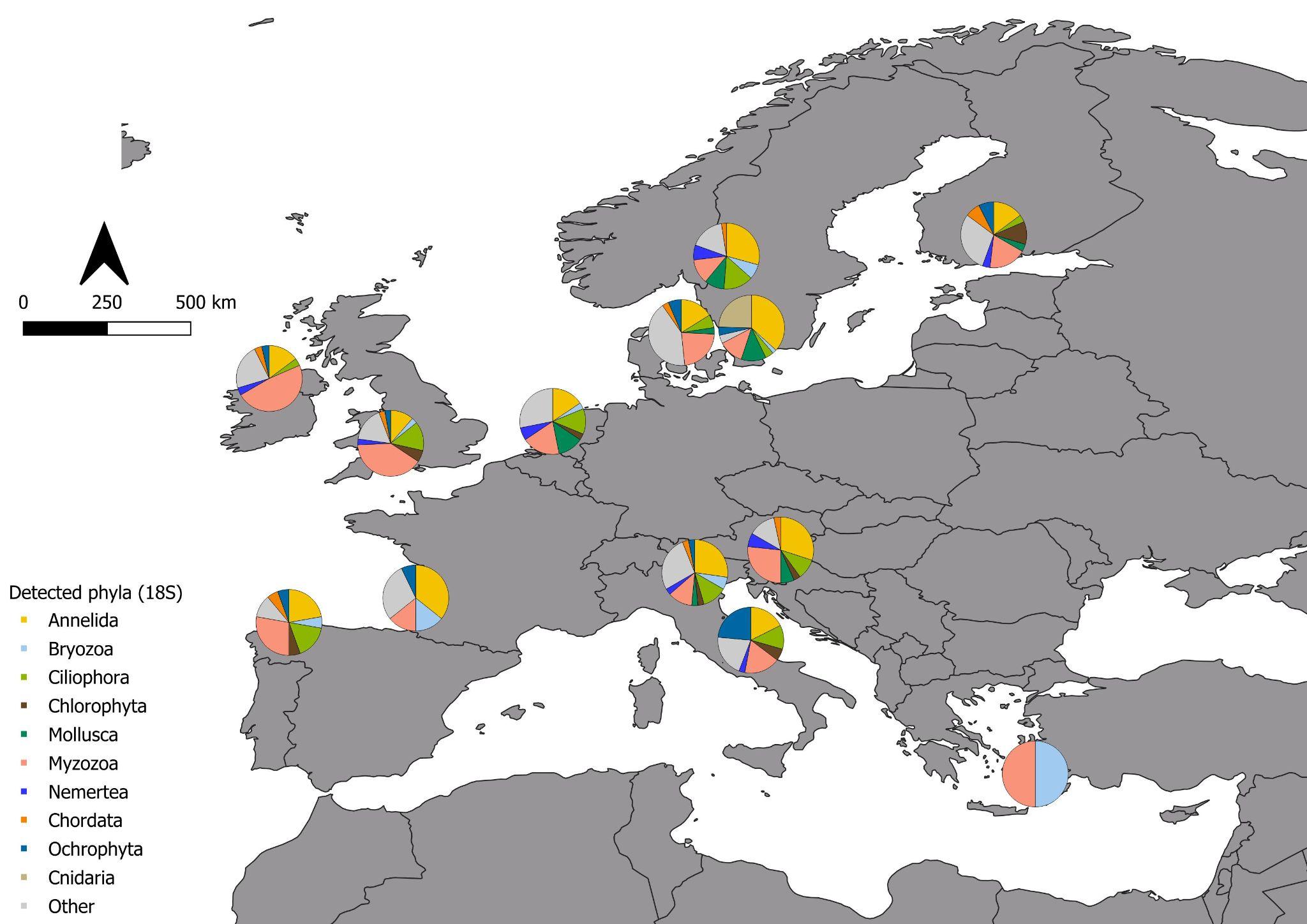


**Supplementary Figure S5.** Geographic distribution of the 10 most abundant phyla (in terms of number of ASVs) detected with COI marker. Differences in sampling effort, deployment duration or sequencing depth have not been accounted for.


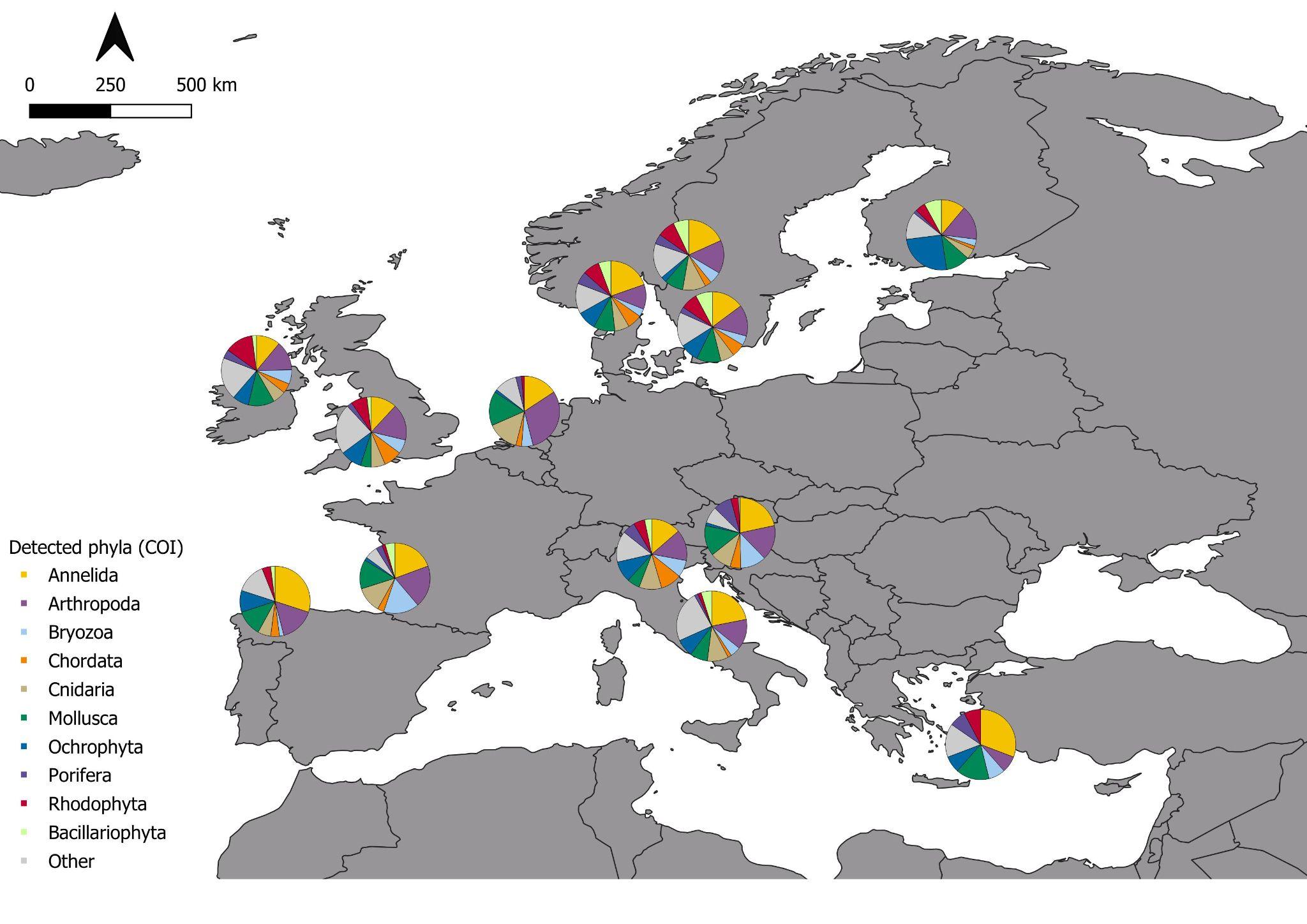

Supplement: Supplementary material 11 — Geographic distribution of the 10 most abundant phyla (for 18S and COI) [file bdj-13-e148981-s011.docx]
